# Community design of the Brooklyn Health Equity Index

**DOI:** 10.1093/haschl/qxae112

**Published:** 2024-09-09

**Authors:** Aimee Afable, Margaret Salisu, Tenya Blackwell, Anthony Divittis, Mark Hoglund, Gwendolyn Lewis, Carla Boutin-Foster, Montgomery Douglas

**Affiliations:** School of Public Health, Downstate Health Sciences University, Brooklyn, NY 11203, United States; Department of Medicine, Downstate Health Sciences University, Brooklyn, NY 11203, United States; Arthur Ashe Institute for Urban Health, Brooklyn, NY 11203, United States; Ambulatory Services, One Brooklyn Health, Brooklyn, NY 11238, United States; School of Public Health, Downstate Health Sciences University, Brooklyn, NY 11203, United States; Ambulatory Services, One Brooklyn Health, Brooklyn, NY 11238, United States; Department of Medicine, Downstate Health Sciences University, Brooklyn, NY 11203, United States; Department of Family and Community Medicine, Downstate Health Sciences University, Brooklyn, NY 11203, United States

**Keywords:** health equity, community-engaged research, quality improvement

## Abstract

Health equity drives quality care. Few reliable metrics that capture patients’ perceptions of health equity exist. We report on the development of a patient-centered metric for health systems change in central Brooklyn, which stands out as an outlier in New York City with a disproportionate burden of poverty, disease, and death. A community-engaged, sequential, mixed-methods research design was used. Qualitative interviews were conducted with 80 community and health care stakeholders across central Brooklyn. Candidate items were derived from qualitative themes and examined for face, interpretive validity, and language. Interitem reliability and confirmatory factor analysis was assessed using data collected via text and automated discharge calls among 368 patients from a local hospital. Qualitative data analysis informed the content of 11 draft questions covering 3 broad domains: trust-building, provider appreciation of social determinants of health, and experiences of discrimination. Psychometric testing resulted in a Cronbach's alpha of 0.774 and led to deletion of 1 item, resulting in a 10-item Brooklyn Health Equity Index (BKHI). The 10-item BKHI is a novel, brief, and reliable measure that captures patients’ perceptions of inequities and offers a real-time measure for health systems and payors to monitor progress toward advancing health equity.

## Introduction

The United States has an escalating health care crisis^1^ where people of color and who are low-income bear a disproportionate burden of disease and death. Two decades after the Institute of Medicine’s (IOM's) seminal 2003 report, *Unequal Treatment: Confronting Racial and Ethnic Disparities in Healthcare*, was published,^2^ the harsh reality of social inequality in the United States is again propelled to the forefront of national conversation. The COVID-19 pandemic led to an aggregate 1.5-year decline in US life expectancy, the largest among affluent peer countries around the world,^3^ and an even more staggering decline in US Black and Hispanic persons’ life expectancy (reaching a 3-year decline).^4^ This pattern, perhaps foreshadowed by the IOM report, came as no surprise for many of us who have dedicated careers to advocating and caring for populations that have been historically marginalized.

According to the World Health Organization (WHO), health inequities are systemic differences in health status of different population groups.^5^ This definition places accountability on systems that have created and sustained unequal practices causing and perpetuating disparities in health and health care. We achieve health equity only when everyone has a just and fair opportunity to attain their full potential for health and well-being.^6,7^

Against this backdrop, patient-centered care and health equity have become major aspirations in national policy and health care payer discussions and initiatives.^8-10^ There is urgency in understanding how different health care systems, particularly those accountable to public funds, are making progress toward achieving health equity, if any. Potential value lies in leveraging hospital quality metrics and surveys, which have been routinely used in the United States. In 2006, the Hospital Consumer Assessment of Healthcare Providers and Systems (HCAHPS) survey was launched. The Centers for Medicare and Medicaid Services (CMS) and Agency for Healthcare Research and Quality (AHRQ) led the development of HCAHPS with the intent to standardize patients’ reports of quality of care and to facilitate dissemination and comparisons of hospital performance metrics to the public.^11,12^

While there was an attempt to identify survey domains from a diverse group of consumers, such surveys were not developed with broad participation of communities that bear the disproportionate burden of disease and death in the United States. The original HCAHPS relied on findings from 16 focus groups in 4 cities across the United States (Phoenix, Los Angeles, Orlando, and Baltimore)^13^; it is notable that several major US cities were excluded, including the largest one, New York City (NYC), despite its growing income inequality^14^ and fragmented health care system.^15,16^

Based on fundamental principles of community-engaged research, lack of community participation in the development of research questions can hamper data-collection efforts because there is no value placed on bidirectional flows of knowledge and benefits^17^ and because of the history of mistrust in the research process and in the health care system more broadly.^18,19^ A history of marginalization can often fuel community perceptions of neglect, divestment, and mistrust, leading to disengagement in the health care system and perpetuating health disparities. There is limited value in research participation to populations who have been repeatedly marginalized by the system.

Response rates for these hospital discharge surveys remain historically low.^20^ Although sparse, there is evidence of significantly lower response rates to selected HCAHPS questions among patients of color, relative to non-Hispanic White patients and among low socioeconomic status (SES) patients relative to affluent patients.^21,22^ Further, acknowledgement of racial/ethnic variation in response rates in studies that report on HCAHPS patient satisfaction is almost nonexistent.^21^ To the extent that patients who respond to hospital discharge surveys are non-Hispanic White, more affluent, or have more trust in the health care system—a form of selection bias—then patient satisfaction may be overestimated. A neglected and serious implication of this bias is that it casts doubt upon the validity of these metrics, and by extension, the meaningfulness of the patient-satisfaction data that exist and any system reform or quality-improvement initiative that the data might inform.^23^ Improving quality of care does not always equate to improving equity in care.^24^ For example, due to advances in health care, between 1990 and 2005, US mortality rates for heart disease, breast cancer, and stroke decreased, but the gap in mortality rates between Black patients and White patients widened.^24^

Practical strategies that can empower health care providers to introduce an equity perspective into real-time quality-improvement initiatives are lacking.^25^ The question remains whether existing standardized surveys designed to measure quality of care and patient satisfaction can be leveraged to provide information on a health care system's progress toward achieving health equity.

Central Brooklyn is one of NYC's historically excluded areas. It stands out as an outlier in NYC with a disproportionate burden of poverty, disease, and mortality. It also has a large concentration of immigrants and Black individuals.^26-29^ The disproportionate burden of disease and mortality in central Brooklyn has persisted throughout the 21st century. For example, the 2001 death rate for all causes was 30% higher in central Brooklyn than for NYC as a whole.^26^ Compared with NYC, central Brooklyn's death rates were also higher for heart disease (10% greater) and cancer (25% greater).^26^ Later data suggest that these patterns have persisted and perhaps worsened. The rate of premature (below age 65 years) deaths for the period 2011–2015 was more than 70% greater in central Brooklyn than for NYC as a whole.^27-29^ Central Brooklyn also saw over 80% more premature heart disease deaths and close to 50% more premature cancer deaths.^27-29^ Finally, a newly released 2024 NY State Department of Health report shows poor overall hospital quality scores across Brooklyn, with the lowest quality scores in majority Black neighborhoods and the largest capacity and access gaps in neighborhoods with a high concentration of Blacks and Hispanics.^30^ As a result, the report cites a trend in Brooklyn residents seeking care elsewhere in more affluent, well-resourced areas of Manhattan.^30^ Based on these data, it is evident that there has been systematic divestment of central Brooklyn, continuing NYC's legacy of “separate and unequal care.”^31^

To reverse this history of systemic marginalization, an intersectoral collaboration representing academics, patient advocacy councils, community leaders, and health care safety-net institutions who serve central Brooklyn came together to develop the Brooklyn Health Equity Index (BKHI). The BKHI is a 10-item health equity metric developed over the course of a 2-year community-engaged, mixed-methods research project. The BKHI was designed to center community voices and lived experiences. We report on the methodology and development of the 10-item BKHI survey.

## Data and methods

### Research team

The BKHI project leadership team consists of faculty and health care leaders of Downstate Health Sciences University, One Brooklyn Health System, and Arthur Ashe Institute for Urban Health. The project was overseen by a community change committee (CCC). The CCC was engaged in all aspects of the research process, including concept and protocol development, outreach, recruitment, data collection, data analysis, dissemination, and evaluation.

A 7-member core research team led by experienced academic and community researchers implemented an exploratory sequential mixed-methods research study in 2 phases.^32^ The core research team was diverse with regard to race and ethnic origins, gender identity, career stage (eg, faculty and students), and health sector (eg, academic researcher vs health care practitioner). Two members of the core team coordinated and facilitated all phase 1 data collection, personally conducting all focus groups and key informant interviews discussed below. By directly engaging with our study participants and building their trust, these 2 core team members gained valuable insight into context and participants’ motivations to ensure that our study participants’ voices are heard and documented.

### Design overview and conceptual framework

**Figure 1.**
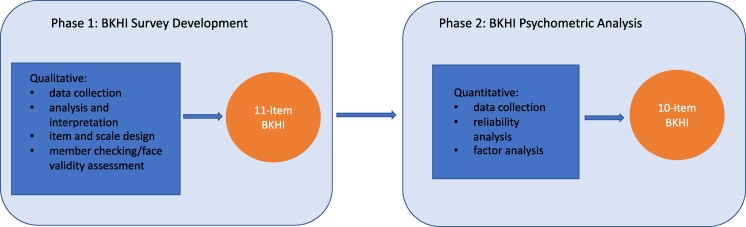
Exploratory sequential mixed-methods design for the Brooklyn Health Equity Index (BKHI).

Guided by principles of community-engaged research,^17^ our research consisted of 2 phases (see Figure 1). In phase 1, we interviewed 62 participants via 10 focus groups (FGs) and 18 key informant interviews (KIIs) representing diverse stakeholders across central Brooklyn—patients, clinicians, community-based organization (CBO) staff, administrators, and medical students. Phase 1 was guided by the equity implementation framework and our working conceptual definition of health equity as stated earlier, which is the idea that health equity is achieved only when everyone has a fair and just opportunity to be as healthy as possible.^7,33^ Our framework recognizes multiple determinants of health operating at the individual, organizational, community, and policy levels. Within each domain, cultural factors, such as medical mistrust or biases, patient–provider interactions, and the social, political, and economic structures and processes play a role in health care equities. From a health systems perspective, there is urgency in identifying and removing structural barriers that pose a threat to health equity and ensure procedural equity.^7,34^ These considerations include understanding and documenting experiences of discrimination, differential treatment, and inclusion in decision-making during the patient–provider encounter. As such, all semi-structured interview guides for this phase included questions addressing these domains a priori.

Consistent with phenomenology studies, phase 1 qualitative data collection was completed when we reached theoretical saturation or when we were no longer obtaining any new significant information. Interpretative phenomenological analysis (IPA) was used as the guiding approach to explore the participants’ lived experiences. The recorded interviews were transcribed and 3 qualitative researchers read the transcripts several times to build familiarity and begin interpreting the data.

This phase was designed to capture and highlight the community's lived experiences and personal narratives, which then directly informed the iterative development of 11 BKHI survey items. A rigorous review of the first iteration of the 11 BKHI items was conducted to assess face and interpretive validity to ensure that our survey items resonate with the community.^35^ First, a series of 4 core research team meetings were held to discuss the language for each item to ensure they captured the personal narratives communicated to us. Second, we conducted member checking^36^ via 2 community FGs where all 11 BKHI items were presented to a subset of community participants (*n* = 10 FG participants) recruited from the original set of FGs and KIIs. The participants were asked to review each item, express their understanding of what the item is measuring, and provide feedback on the wording, order of items, and overall appearance of the BKHI items. Third, a web-based survey that listed each of the original 11 BKHI items was distributed to the 15-member BKHI project leadership team to query their opinions on the face validity of each BKHI item.

In phase 2, we performed reliability and confirmatory factor analysis to finalize the BKHI item selection. This psychometric testing was conducted based on data collected via SMS (Short Message Service) text and robocall discharge surveys from *n* = 368 patients in key clinical settings at the 3 facilities of a major central Brooklyn safety-net health care system in Brooklyn. Data were analyzed using SPSS Statistics (version 29; IBM Corporation). Categorical data were characterized by frequency and percentage. Continuous data were characterized by mean and SD. Responses to BKHI items required respondents to indicate their degree of agreement to the item according to the following categories: 1 = agree, 2 = disagree, and 3 = unsure. In order to create a Likert-type scale, items were recoded as 1 = agree, 2 = unsure, and 3 = disagree. Where necessary, items were recoded so that high scores consistently indicated high levels of health equity and low scores indicated high levels of health inequity. Scores ranged from 11 (low levels health equity) to 33 (high levels of health equity). Factor analysis was conducted using the extraction method of principal components analysis with a varimax rotation.

## Results

### Phase 1

#### Results of qualitative data analysis

The qualitative data revealed 3 primary themes: trust, discrimination, and appreciation of social determinants of health (SDOHs). Each theme comprised subthemes as follows: For trust, the subthemes included (1) confidence in the health care professional, (2) provider empathy, and (3) active participation in health care decisions. Regarding discrimination, the subthemes involved (1) racism and identity as well as (2) stigma related to diagnosis, disease state, and pain management. In addition, there was a perception of divestment in the hospital facilities in central Brooklyn as articulated by 1 of our participants (highlighted in Table 1; last quote). Last, for SDOHs, the key subtheme was the acknowledgment by providers that patients encounter competing priorities acting as barriers to care, such as housing instability and food insecurity.^37^ A total of 907 codes were identified in the transcripts. An analysis of the codes revealed that 35% were related to distrust while 55% of the codes were directly related to themes of discrimination. Social determinants of health accounted for 10% of the codes.^37^ These data and themes were then used to develop 11 survey items found below (Table 1).

**Table 1.** How the themes from the qualitative data informed each BKHI item.

Theme	Items
Social determinants of health: Recognition by providers that patients face competing priorities that may serve as barriers to care (housing instability, food insecurity, etc) Example quote: “I was saying that the med school does try to encourage education to help us understand the social determinants of health… but I think there is this disconnect. Where we can kind of help patients reach that next step, so you know you might tell them, ‘Here's a diet you have to follow for diabetes or blood pressure,’ but then you can't actually help them go one step further and realize that maybe they don't have access to those particular food items that they need, or they won't be able to get the medications that they need because the pharmacy is too far. So we might be educated about it but we're not given enough tools to bridge that final gap to make sure that we can actually help remedy the problem or make it a little better.” (medical student)	1. The provider and care team asked about other aspects of my life when helping me make health care decisions. 2. My care team recognized how hard it can be to follow their medical advice in everyday life.
Trust: Clear communication between patient and provider Provider empathy Active partner in health care decisions Trust in the professionals providing care Respect for patients’ time Example quote: “Well, I don’t trust doctors at all, just for the main fact that when my daughter was 10 years old, we were sent to see a specialist. They said they thought she had a hernia. The doctor came into the room. He didn’t even examine my daughter; he just gave us the deed for the surgery, and I was like, ‘You didn’t even examine her, so how are you so sure that it is a hernia?’” (patient) “I think a lot of the time it was because there was a lack of transparency. They weren’t fully explained things, and they were like, things are just happening to them without them being involved in the discussion of like, whenever we’re talking about informed consent, we have to tell a patient all the different options, including no treatment, but that doesn’t happen all the time, and they’re kind of told, ‘This is what we’re going to do, this is what's going to happen to you,’ and they’re not really told all the risks and benefits—are not told all the options. So, I think that inherently brings distrust, and it's completely understandable.” (provider)	3. I feel I can trust the provider and staff in this facility. 4. I participated as much as I wanted in making decision regarding my health care. 5. I was happy with the way my provider and/or staff listened to me and answered my questions. 6. Staff made me feel welcomed. 7. I experienced long wait times.
	
Discrimination (individual and institutional): Marginalized populations Disrespect/disregard Stigma associated with medical conditions/needs History of divestment in hospital infrastructure Example quote: “He said, ‘You’re fat, you’re overweight.’ I also have COPD and asthma, and I’m a diabetic. You know, when you take a lot of steroids, you gain weight, so they fix one problem, but some medications cause other problems, right? So, when I go to doctors, they tell me, ‘Oh, you’re just fat. Have you ever thought about getting surgery?’ What?” (patient) “Recently, I was at [Institution], I saw a Haitian man who couldn’t communicate with the nurses being treated badly. The staff neglected him because of his inability to speak proper English, and nobody was helping him. I wondered, ‘Why can’t they get somebody who can speak Haitian to come and talk to him and see what his problem is?’ But they just left him there.” (patient) “Go across one of the bridges or tunnels into Manhattan. You'll notice that every single one of the major medical institutions there have some rich person name on a building. Billionaires who are grateful patients and then commit a huge amount of money to the institutions where they are treated. Those patients don't come to central Brooklyn for care…. We have a very hard time attracting donors who are willing to give us $100 million to $200 million to build fancy buildings and fancy facilities. So there is a significant inherent bias in the way this system in this country is constructed. The way it's paid for, and the way hospitals like ours are sustained.” (hospital administrator)	8. I felt I was treated unfairly or disrespected because of my race, ethnicity, age, language, sexual orientation, gender identity, or gender expression. 9. There were times I felt shamed/blamed because of my physical condition. 10. I felt dismissed or disregarded by the provider and/or staff because of my physical condition. 11. The state of the facility and the medical equipment made me wonder whether I was getting the best possible care.

#### Face and interpretive validity analysis

During the community listening sessions, participants were able to accurately identify the themes that the items were designed to measure. The impassioned response from participants during the listening sessions underscored the need to amplify the voices of patients who have historically felt disenfranchised by the health care system. The researchers found that the participants’ identification of the themes for each item aligned with their own. Additionally, the participants suggested changes to the order of the items. Results from the web-based survey administered to the entire BKHI Grant Implementation Team were also consistent with findings from the community listening sessions. All themes identified in the 11-item survey were validated or accurately identified based on the responses from this 12-member project leadership team.

### Phase 2

Psychometric testing was performed based on BKHI data collected from a sample of 368 patients discharged from a major health care safety-net system in central Brooklyn. Response rates ranged from 7% for surveys administered via robocall to 27% for surveys administered via SMS text message. The sample was diverse with regard to hospital unit visited or site of discharge, with 42% representing ambulatory care, 45% representing the emergency department, 6% representing inpatient units, and 8% representing maternity care units. The majority of the sample was Black (86%), consistent with the racial/ethnic group distribution of the central Brooklyn population; 69% were female; and the age range was 18–93 years old (mean age = 55 years).

Based on the 11-item survey, reliability was established using an internal consistency analysis yielding a Cronbach's alpha of 0.774. Principal components analysis yielded a 2-factor solution. Combined, the 2 factors accounted for 52% of the total variance. Factor 1 is composed of 6 items that relate primarily to the relationship between the provider and patient. Themes include the following: satisfaction with communication, staff consideration of SDOH issues in health practices, and trust for the provider and staff. Factor 2 is composed of 5 items that relate to a negative patient experience with themes that included unfair treatment based on group identity (eg, race, ethnicity, etc), feeling dismissed by the provider team, and stigma associated with medical condition (see Table 2 for factor loadings.). The item “I experienced long wait times” did not load strongly on either factor. In addition, the item was the least unique to the BKHI and covered in other patient satisfaction surveys (eg, HCAHPS). The item was eliminated from the final version of the BKHI, resulting in a 10-item survey with possible scores ranging from 10 to 30 (see Table 3).

**Table 2.** Factor analysis: item loading.

Factor 1		Factor 2	
Item	Factor loading	Item	Factor loading
Staff made me feel welcomed.	0.676	I experienced long wait times.	0.428
I was happy with the way my provider and/or staff listened to me and answered my questions.	0.781	I felt I was treated unfairly or disrespected because of my race, ethnicity, etc.	0.714
The provider and care team asked about other aspects of my life when helping me make health care decisions.	0.710	There were times I felt shamed/blamed because of my physical condition.	0.750
My care team recognized how hard it can be to follow their medical advice in everyday life.	0.559	I felt dismissed or disregarded by the provider and/or staff about my medical condition	0.772
I felt I can trust the provider and staff in this facility.	0.776	The state of the facility and the medical equipment made me wonder whether I was getting the best possible care.	0.629
I participated as much as I wanted in making decisions regarding my health.	0.685		

*n* = 368 survey respondents.

**Table 3.** Final Brooklyn Health Equity Index (BKHI) and Recommended Order and Scoring Strategy.

Introductory Script: The Brooklyn Health Equity Index is a brief, voluntary survey. Your responses to the survey will help us improve health equity in our community. Please review the below statements and think about your experience during your last clinic visit, emergency visit or hospital stay. If you agree, enter “1.” If you disagree, enter “2.” If you are unsure, enter “3.”		
Item	Original score	Recode
1. I felt I was treated unfairly or disrespected because of my race, ethnicity, language, age, disability, sexual orientation, gender identity, or gender expression.	1 = Agree	1 = Agree
	2 = Disagree	2 = Unsure
	3 = Unsure	3 = Disagree
2. Staff made me feel welcomed.	1 = Agree	1 = Disagree
	2 = Disagree	2 = Unsure
	3 = Unsure	3 = Agree
3. I was happy with the way my provider and/or staff listened to me and answered my questions.	1 = Agree	1 = Disagree
	2 = Disagree	2 = Unsure
	3 = Unsure	3 = Agree
4. The provider and care team asked about other aspects of my life when helping me make health care decisions (stressful life experiences such as concerns about mental health, financial problems, housing issues, transportation, etc).	1 = Agree	1 = Disagree
	2 = Disagree	2 = Unsure
	3 = Unsure	3 = Agree
5. I participated as much as I wanted in making decisions regarding my health care.	1 = Agree	1 = Disagree
	2 = Disagree	2 = Unsure
	3 = Unsure	3 = Agree
6. There were times I felt shamed/blamed because of my physical condition (body weight, diagnosis, need for pain medication).	1 = Agree	1 = Agree
	2 = Disagree	2 = Unsure
	3 = Unsure	3 = Disagree
7. My care team recognized how hard it can be to follow their medical advice in everyday life.	1 = Agree	1 = Disagree
	2 = Disagree	2 = Unsure
	3 = Unsure	3 = Agree
8. I felt dismissed or disregarded by the provider and/or staff about my medical condition (levels of pain, symptoms, illness).	1 = Agree	1 = Agree
	2 = Disagree	2 = Unsure
	3 = Unsure	3 = Disagree
9. The state of the facility and the medical equipment made me wonder whether I was getting the best possible care.	1 = Agree	1 = Agree
	2 = Disagree	2 = Unsure
	3 = Unsure	3 = Disagree
10. I feel I can trust the provider and staff of this facility.	1 = Agree	1 = Disagree
	2 = Disagree	2 = Unsure
	3 = Unsure	3 = Agree

BKHI^©^2023, The Research Foundation for the State University of New York. All rights reserved.

## Discussion

Our goal was to develop a health equity metric for systems change. Our 2-year community-engaged research led to the development of a 10-item BKHI. Psychometric analysis yielded high reliability and a 2-factor solution. The first factor captured a construct that had high correlations to the items that were categorized in the SDOH and trust domains (per phase 1). The second factor captured perceived interpersonal and institutional discrimination, which were prominent themes revealed in our phase 1 qualitative data findings^37^; it is notable that the distinction in the multiple levels of discrimination is consistent with Jones’ multiple levels of racism framework.^38^ In the development and final selection of the BKHI items, in order to maintain fidelity to the principles of community-engaged research we had to carefully balance the results from phase 1 listening sessions and phase 2 psychometric analysis. Rather than aiming for parsimony, we opted to preserve items because of their meaning to the community, despite evidence of redundancy or similar factor loadings, resulting in the exclusion of 1 single item from our original draft set of items from phase 1.

There is a long history of studying and measuring SDOHs, perceived discrimination, and trust in the provider/health care system in the public health scholarship.^38-44^ These concepts have long been valued by the public health community and our work is part of a body of work that demonstrates that these concepts also resonate with the lived experiences of communities of color or communities that have been historically marginalized.^45-47^ What makes the BKHI novel is that (1) it brings together these concepts of discrimination, trust, and appreciation of SDOHs in 1 measure that is intended to be administered in diverse clinical settings; (2) it is distinct from existing HCAHPS patient experience surveys that have been universally adopted by US health care systems; and (3) it was designed using the principles of community-engaged research^17^ with a single purpose to amplify the voices of members of the central Brooklyn community who have experienced a history of marginalization. With regard to HCAHPS, the survey is composed of 29 items and captures the following core domains: communication with nurses, communication with doctors, responsiveness of hospital staff, communication about medicines, discharge information, care transition, cleanliness of the hospital environment, quietness of the hospital environment, overall rating of the hospital, and recommendation of the hospital.^48^ These items do not directly address patient perceptions of discrimination and disrespect, trust, provider empathy, and provider recognition of competing daily priorities/survival needs of patients that may serve as barriers to care (ie, food insecurity, housing instability, and childcare). Our novel BKHI instrument addresses all these concerns.

In order to promote health equity, health systems must take steps to identify and remove structural barriers that pose a threat to health equity. The BKHI provides health systems with real-time data that can be used to identify potential barriers to achieving health equity, including discrimination, lack of trust, and lack of provider appreciation of SDOHs. For example, there is a robust evidence base on the association between perceived discrimination and poor quality of life and physical and mental health.^49-53^ Therefore, experiences of discrimination in the health care setting can ultimately limit the opportunity to achieve the best health possible. The BKHI can identify the prevalence of perceived discrimination and institutional racism.^38^ Moreover, Chandra et al^34^ delineated equity into distinct forms and described procedural equity as perceived fairness of processes or procedures such as trust in the health system. This also includes representation and inclusion in decision-making. The BKHI provides data on patients’ perceptions of their inclusion in the decision-making process. Therefore, health care systems can use real-time data from the BKHI to identify with greater precision gaps in care to inform the design of professional development training or quality-improvement initiatives.^54^

### Implications for policy and health care reform

The development and introduction of the BKHI to the public is timely. It aligns with national policy priorities that focus on health equity and patient experience and with ongoing health care reform initiatives in New York State. New health care equity standards were introduced into the Joint Commission's hospital, ambulatory, and behavioral health care accreditation programs as of January 2023.^55^ The CMS is finalizing a 2024 Hospital Value-Based Purchasing Program that rewards hospitals with higher payments based on SDOH reporting.^56^ Our novel BKHI instrument can be considered as part of the Health Equity Regional Planning Investment plan outlined in the New York State Department of Health, Office of Health Insurance Program Conceptual Framework for the 1115 Waiver Demonstration.^10^ The plan calls for a statewide and regionally specific set of health equity–specific quality-improvement measures to achieve regional priorities. Thus, the BKHI can be used to set baseline standards for equity and to monitor progress prospectively.

### Limitations and considerations for more generalized applications

The BKHI survey was developed and grounded in the experiences of people living in central Brooklyn and may not be representative of the experiences of other populations who have been historically marginalized or who use other types of health care systems. However, we do believe the concepts may resonate in settings and populations outside of central Brooklyn because they speak to universal concerns of trust building between provider and patient, provider empathy, clear communication, and respect and appreciation of differences based on self-identity. The BKHI survey was psychometrically tested based on a sample patient population that represented a diverse range of hospital settings, including ambulatory, emergency department, maternity care, and inpatient settings. Therefore, there is potential for wide applicability to other settings, including federally qualified health centers, health care payer plan members, as well as health care system diagnostic and treatment centers.

### Comment on BKHI response rate and recommended use

The BKHI data and response rates presented here were collected based on post-visit and post-discharge surveys. It is important to note that our SMS response rate of 27% is comparable to response rates for other NYC surveys administered at the population level^57^ and within range, and in many cases, exceeds published response rates for hospital-administered patient experience surveys.^22,58-66^ For example, Roberts et al^22^ analyzed May 2019 response rates for the Clinician and Group (CG)–CAHPS survey of patient experience for outpatient clinic visits at a large academic health system in New Jersey and found an overall response rate of 12.8%; they found an SES gradient in survey response rates ranging from 6.3% in the municipality with the lowest median household income to 17.7% in the municipality with the highest median annual household income.

We recommend that BKHI items are administered via SMS message within 1 week following an outpatient visit or hospital discharge. The response rates were based on the following post-visit/discharge time frame and strategy—(1) first contact: SMS (text) message 1 day after visit/discharge; (2) second contact: SMS message second day after visit/discharge; and (3) third contact: automated voice phone call on the fourth day after visit/discharge. Finally, if an SMS message could not be sent at first contact due to lack of a smart phone or wrong number, then the patient was sent an automated voice call on the second day. Table 3 lists the recommended (1) order of the BKHI items when administering the BKHI and (2) scoring strategy.

## Conclusion

The final short 10-item BKHI questionnaire is novel and easy to use by health care staff and stakeholders. We envision that the BKHI can be used as a tool or quantitative metric by health care system leadership and health care payers to monitor progress toward achieving health equity and to advocate for quality-improvement initiatives. The BKHI, by design, has the potential to drive transformation at the patient–provider level by providing real-time information on patient experiences within the domains of trust, discrimination/disrespect, and provider acknowledgment of SDOHs, and at the systems level by providing an aggregate measure of health equity. Our long-term vision is to be able to evaluate the impact of routine health care system use of the BKHI on patient experience and on health system performance in making progress toward health equity. These values have been advanced by authoritative health agencies since the turn of the 21st century.^2,67-69^

## Supplementary Material

qxae112_Supplementary_Data

## References

[1] Wadhera RK , Dahabreh IJ. The US health equity crisis—an economic case for a moral imperative? JAMA. 2023;329(19):1647–1649. 37191712 10.1001/jama.2023.4018

[2] Institute of Medicine Committee . Unequal Treatment: Confronting Racial and Ethnic Disparities in Health Care. National Academies Press (US); 2003. 25032386

[3] Woolf SH , Masters RK, Aron LY. Changes in life expectancy between 2019 and 2020 in the US and 21 peer countries. JAMA Netw Open. 2022;5(4):e227067. 35416991 10.1001/jamanetworkopen.2022.7067 PMC9008499

[4] Arias E, Tejada-Vera B, Ahmad F, Kochanek KD. Provisional life expectancy estimates for 2020. Accessed February 19, 2024. https://www.cdc.gov/nchs/data/vsrr/vsrr015-508.pdf

[5] World Health Organization. Health equity and their causes. Accessed February 19, 2024. https://www.who.int/news-room/facts-in-pictures/detail/health-inequities-and-their-causes

[6] World Health Organization. Health equity. Accessed February 19, 2024. https://www.who.int/health-topics/health-equity#tab=tab_1

[7] Braveman P , Arkin E, Orleans T, Proctor D, Plough A. What is health equity? Robert Wood Johnson Foundation; 2020. Retrieved November 4, 2017.

[8] Centers for Medicare and Medicaid Services . CMS Framework for Health Equity. CMS; 2023. Accessed October 20, 2023. https://www.cms.gov/priorities/health-equity/minority-health/equity-programs/framework

[9] New York State Department of Health. A plan to transform the Empire State’s Medicaid program. Accessed February 19, 2024. https://www.health.ny.gov/health_care/medicaid/redesign/docs/mrtfinalreport.pdf

[10] New York State Department of Health Office of Health Insurance Programs. 1115 Waiver Demonstration: Conceptual framework. A federal-state partnership to address health disparities exacerbated by the COVID-19 pandemic. Accessed January 14, 2024. https://www.health.ny.gov/health_care/medicaid/redesign/2021/2021-08_1115_waiver_concept_paper.htm#_TOC_250011

[11] Goldstein E , Farquhar M, Crofton C, Darby C, Garfinkel S. Measuring hospital care from the patients’ perspective: an overview of the CAHPS Hospital Survey development process. Health Serv Res. 2005;40(6 Pt 2):1977–1995. 16316434 10.1111/j.1475-6773.2005.00477.x PMC1361247

[12] Giordano LA , Elliott MN, Goldstein E, Lehrman WG, Spencer PA. Development, implementation, and public reporting of the HCAHPS survey. Med Care Res Rev. 2010;67(1):27–37. 19638641 10.1177/1077558709341065

[13] Sofaer S , Crofton C, Goldstein E, Hoy E, Crabb J. What do consumers want to know about the quality of care in hospitals? Health Serv Res. 2005;40(6 Pt 2):2018–2036. 16316436 10.1111/j.1475-6773.2005.00473.x PMC1361244

[14] Stefanos C . New York is rebounding for the rich. Nearly everyone else is struggling; New York. September 28, 2023. Accessed January 14, 2024. https://www.nytimes.com/2023/09/28/nyregion/nyc-income-gap-wages.html. 2023.

[15] Dickman SL , Himmelstein DU, Woolhandler S. Inequality and the health-care system in the USA. Lancet. 2017;389(10077):1431–1441. 28402825 10.1016/S0140-6736(17)30398-7

[16] Schrag D , Xu F, Hanger M, Elkin E, Bickell NA, Bach PB. Fragmentation of care for frequently hospitalized urban residents. Med Care. 2006;44(6):560–567. 16708005 10.1097/01.mlr.0000215811.68308.ae

[17] Israel BA , Schulz AJ, Parker EA, Becker AB. Review of community-based research: assessing partnership approaches to improve public health. Annu Rev Public Health. 1998;19(1):173–202. 9611617 10.1146/annurev.publhealth.19.1.173

[18] Wolinetz CD , Collins FS. Recognition of research participants’ need for autonomy: remembering the legacy of Henrietta Lacks. JAMA. 2020;324(11):1027–1028. 32930765 10.1001/jama.2020.15936

[19] Brandon DT , Isaac LA, LaVeist TA. The legacy of Tuskegee and trust in medical care: is Tuskegee responsible for race differences in mistrust of medical care? J Natl Med Assoc. 2005;97(7):951–956. 16080664 PMC2569322

[20] Schopf AC , Vach W, Jakob M, Saxer F. Routine patient surveys: patients’ preferences and information gained by healthcare providers. PLoS One. 2019;14(8):e0220495. 31369612 10.1371/journal.pone.0220495 PMC6675389

[21] Abdelgadir J , Ong E, Abdalla S, et al Demographic factors associated with patient-reported outcome measures in pain management. Pain Physician. 2020;23(1):17–24. 32013275

[22] Roberts BW , Yao J, Trzeciak CJ, Bezich LS, Mazzarelli A, Trzeciak S. Income disparities and nonresponse bias in surveys of patient experience. J Gen Intern Med. 2020;35(7):2217–2218. 32006345 10.1007/s11606-020-05677-6 PMC7351907

[23] Mazor KM , Clauser BE, Field T, Yood RA, Gurwitz JH. A demonstration of the impact of response bias on the results of patient satisfaction surveys. Health Serv Res. 2002;37(5):1403–1417. 12479503 10.1111/1475-6773.11194 PMC1464019

[24] Dzau VJ , Mate K, O’Kane M. Equity and quality—improving health care delivery requires both. JAMA. 2022;327(6):519–520. 35060998 10.1001/jama.2022.0283

[25] Hausmann LRM , Lamorte C, Estock JL. Understanding the context for incorporating equity into quality improvement throughout a National Health Care System. Health Equity. 2023;7(1):312–320. 37284535 10.1089/heq.2023.0009 PMC10240324

[26] Karpati A , Lu X, Mostashari F, Thorpe L, Frieden TR. The Health of Central Brooklyn. New York. Accessed October 26, 2023. https://www.nyc.gov/assets/doh/downloads/pdf/data/2003nhp-brooklyna.pdf. 2003.

[27] Hinterland K , Naidoo M, King L, et al Brooklyn Community District 3: Bedford Stuyvesant. New York. Accessed October 26, 2023. https://www.nyc.gov/assets/doh/downloads/pdf/data/2018chp-bk3.pdf. 2018.

[28] Hinterland K , Naidoo M, King L, et al Brooklyn Community District 16: Brownsville. New York. Accessed October 26, 2023. https://www.nyc.gov/assets/doh/downloads/pdf/data/2018chp-bk16.pdf. 2018.

[29] Hinterland K , Naidoo M, King L, et al Brooklyn Community District 8: Crown Heights and Prospect Heights. New York. Accessed October 26, 2023. https://www.nyc.gov/assets/doh/downloads/pdf/data/2018chp-bk8.pdf. 2018.

[30] NYS Department of Health, Report on the New York State Department of Health’s Study of Healthcare System Inequities and Perinatal Access in Brooklyn, New York . Accessed February 11, 2024. https://www.health.ny.gov/press/reports/docs/brooklyn_perinatal_access_report.pdf.

[31] Calman NS , Golub M, Ruddock C, Le L, Hauser D; Action Committee of the Bronx Health REACH Coalition. Separate and unequal care in New York City. J Health Care Law Policy. 2006;9(1):105–120. 17165226

[32] Creswell JW , Poth CN. Qualitative Inquiry and Research Design Choosing among Five Approaches. 4th ed. Sage Publications, Inc; 2018.

[33] Woodward EN , Singh RS, Ndebele-Ngwenya P, Melgar Castillo A, Dickson KS, Kirchner JE. A more practical guide to incorporating health equity domains in implementation determinant frameworks. Implement Sci Commun. 2021;2(1):61. 34090524 10.1186/s43058-021-00146-5 PMC8178842

[34] Chandra A , Martin LT, Acosta JD, et al Equity as a guiding principle for the public health data system. Big Data. 2022;10(S1):S3–S8. 36070506 10.1089/big.2022.0204 PMC9508440

[35] McKim C . Meaningful member-checking: a structured approach to member-checking. Am J Qual Res. 2023;7(2):41–52.

[36] Varpio L , Ajjawi R, Monrouxe LV, O'Brien BC, Rees CE. Shedding the Cobra effect: problematising thematic emergence, triangulation, saturation and member checking. Med Educ. 2017;51(1):40–50. 27981658 10.1111/medu.13124

[37] Salisu M , Blackwell TM, Lewis G, et al Community perceptions of health equity: a qualitative study. J Prim Care Community Health. 2023;14:21501319231211439. 37978842 10.1177/21501319231211439 PMC10657528

[38] Jones CP . Levels of racism: a theoretic framework and a gardener's tale. Am J Public Health. 2000;90(8):1212–1215. 10936998 10.2105/ajph.90.8.1212 PMC1446334

[39] Williams DR , Yan Y, Jackson JS, Anderson NB. Racial differences in physical and mental health: socio-economic status, stress and discrimination. J Health Psychol. 1997;2(3):335–351. 22013026 10.1177/135910539700200305

[40] Braveman P , Egerter S, Williams DR. The social determinants of health: coming of age. Annu Rev Public Health. 2011;32(1):381–398. 21091195 10.1146/annurev-publhealth-031210-101218

[41] Marmot MG , Smith GD, Stansfeld S, et al Health inequalities among British civil servants: the Whitehall II study. Lancet. 1991;337(8754):1387–1393. 1674771 10.1016/0140-6736(91)93068-k

[42] Kawachi I , Berkman L. Social cohesion, social capital, and health. In: Berkman L, Kawachi I, eds. Social Epidemiology. Oxford University Press; 2000:174–190.

[43] Hall MA , Dugan E, Zheng B, Mishra AK. Trust in physicians and medical institutions: what is it, can it be measured, and does it matter? Milbank Q. 2001;79(4):613–639, v. 11789119 10.1111/1468-0009.00223 PMC2751209

[44] Peek ME , Nunez-Smith M, Drum M, Lewis TT. Adapting the everyday discrimination scale to medical settings: reliability and validity testing in a sample of African American patients. Ethn Dis. 2011;21(4):502–509. 22428358 PMC3350778

[45] Muraskin A. , 'Irth' hospital review app aims to take the bias out of giving birth. Accessed February 19, 2024. https://www.npr.org/sections/health-shots/2023/10/13/1205333159/irth-hospital-review-app-aims-to-take-the-bias-out-of-giving-birth. 2023.

[46] Scharff DP , Mathews KJ, Jackson P, Hoffsuemmer J, Martin E, Edwards D. More than Tuskegee: understanding mistrust about research participation. J Health Care Poor Underserved. 2010;21(3):879–897. 20693733 10.1353/hpu.0.0323 PMC4354806

[47] Damle M , Wurtz H, Samari G. Racism and health care: experiences of Latinx immigrant women in NYC during COVID-19. SSM Qual Res Health. 2022;2:100094. 35578651 10.1016/j.ssmqr.2022.100094 PMC9095080

[48] Hospital Consumer Assessment of Healthcare Providers and Systems. CAHPS© Hospital Survey. Accessed January 14, 2024. https://hcahpsonline.org/#AboutTheSurvey

[49] Hausmann LR , Jeong K, Bost JE, Ibrahim SA. Perceived discrimination in health care and health status in a racially diverse sample. Med Care. 2008;46(9):905–914. 18725844 10.1097/MLR.0b013e3181792562 PMC3424509

[50] Piette JD , Bibbins-Domingo K, Schillinger D. Health care discrimination, processes of care, and diabetes patients’ health status. Patient Educ Couns. 2006;60(1):41–48. 16332469 10.1016/j.pec.2004.12.001

[51] Mays VM , Jones AL, Delany-Brumsey A, Coles C, Cochran SD. Perceived discrimination in health care and mental health/substance abuse treatment among Blacks, Latinos, and Whites. Med Care. 2017;55(2):173–181. 27753743 10.1097/MLR.0000000000000638 PMC5233585

[52] Williams DR , Lawrence JA, Davis BA. Racism and health: evidence and needed research. Annu Rev Public Health. 2019;40(1):105–125. 30601726 10.1146/annurev-publhealth-040218-043750 PMC6532402

[53] Paradies Y , Ben J, Denson N, et al Racism as a determinant of health: a systematic review and meta-analysis. PLoS One. 2015;10(9):e0138511. 26398658 10.1371/journal.pone.0138511 PMC4580597

[54] Melino K . Structural competency in health care. Nurs Clin North Am. 2022;57(3):433–441. 35985730 10.1016/j.cnur.2022.04.009 PMC9300050

[55] The Joint Commission. Health Care Equity Certification. Accessed February 19, 2024. https://www.jointcommission.org/our-priorities/health-care-equity/

[56] CMS. New CMS Rule Promotes High-Quality Care and Rewards Hospitals that Deliver High-Quality Care to Underserved Populations. Accessed February 19, 2024. https://www.cms.gov/newsroom/press-releases/new-cms-rule-promotes-high-quality-care-and-rewards-hospitals-deliver-high-quality-care-underserved

[57] NYC Health. Community Health Survey Methodology. Accessed February 24, 2024. https://www.nyc.gov/site/doh/data/data-sets/community-health-survey-methodology.page

[58] Zhang T , Schneider MB, Weir TB, et al Response bias for Press Ganey ambulatory surgery surveys after knee surgery. J Knee Surg. 2023;36(10):1034–1042. 35817060 10.1055/s-0042-1748896

[59] Tyser AR , Abtahi AM, McFadden M, Presson AP. Evidence of non-response bias in the Press-Ganey patient satisfaction survey. BMC Health Serv Res. 2016;16(a):350. 27488567 10.1186/s12913-016-1595-z PMC4972948

[60] Malpani R , Adrados M, Mercier MR, et al Characteristics and predictors of HCAHPS nonresponse after spine surgery. Spine (Phila Pa 1976). 2020;45(8):E448–E456. 31609883 10.1097/BRS.0000000000003287 PMC7113123

[61] Henry LE , Zhang T, Aneizi A, et al Perioperative opioid use and Press Ganey patient satisfaction scores after anterior cruciate ligament reconstruction. J Orthop. 2021;27:84–91. 34588743 10.1016/j.jor.2021.09.003 PMC8453184

[62] Compton J , Glass N, Fowler T. Evidence of selection bias and non-response bias in patient satisfaction surveys. Iowa Orthop J. 2019;39(1):195–201. 31413694 PMC6604521

[63] Ahmed AS , Kim RL, Ramsamooj H, Roberts M, Downes K, Mir HR. Patient perception of pain control (not opiate amount) affects hospital consumer assessment of healthcare providers and systems and Press Ganey satisfaction scores after orthopaedic trauma. J Am Acad Orthop Surg. 2021;29(7):301–309. 33443382 10.5435/JAAOS-D-20-00069

[64] Mercier MR , Galivanche AR, David WB, et al Hospital Consumer Assessment of Healthcare Providers and Systems survey response rates are significantly affected by patient characteristics and postoperative outcomes for patients undergoing primary total knee arthroplasty. PLoS One. 2021;16(9):e0257555. 34582475 10.1371/journal.pone.0257555 PMC8478166

[65] Dad T , Tighiouart H, Fenton JJ, et al Evaluation of non-response to the In-Center Hemodialysis Consumer Assessment of Healthcare Providers and Systems (ICH CAHPS) survey. BMC Health Serv Res. 2018;18(1):790. 30340585 10.1186/s12913-018-3618-4 PMC6194668

[66] Abdelgadir J , Ong EW, Abdalla SM, et al Demographic factors associated with patient-reported outcome measures in pain management. Pain Physician. 2020;23(1):17–24. 32013275

[67] Alley DE , Asomugha CN, Conway PH, Sanghavi DM. Accountable health communities–addressing social needs through Medicare and Medicaid. N Engl J Med. 2016;374(1):8–11. 26731305 10.1056/NEJMp1512532

[68] Arend J , Tsang-Quinn J, Levine C, Thomas D. The patient-centered medical home: history, components, and review of the evidence. Mt Sinai J Med. 2012;79(4):433–450. 22786733 10.1002/msj.21326

[69] Berwick DM . A user's manual for the IOM's ‘quality chasm’ report. Health Aff (Millwood). 2002;21(3):80–90. 12026006 10.1377/hlthaff.21.3.80

